# Historical biogeography of the neotropical Diaptomidae (Crustacea: Copepoda)

**DOI:** 10.1186/1742-9994-11-36

**Published:** 2014-05-01

**Authors:** Gilmar Perbiche-Neves, Daniel Previattelli, Marcio R Pie, Andressa Duran, Eduardo Suárez-Morales, Geoffrey A Boxshall, Marcos G Nogueira, Carlos EF da Rocha

**Affiliations:** 1 Departamento de Zoologia, Universidade de São Paulo – USP, IB, Rua do Matão, travessa 14, n. 321, São Paulo, SP CEP 05508-900, Brazil; 2 Universidade Federal do Paraná, Departamento de Zoologia, Laboratório de Dinâmica Evolutiva e Sistemas Complexos, Jardim das Américas, Curitiba, Paraná, CEP 81531-990, Brazil; 3 El Colegio de la Frontera Sur (ECOSUR), Unidad Chetumal, Av. Centenario Km 5.5, Chetumal, Quintana Roo 77014, Mexico; 4 Department of Life Sciences, The Natural History Museum – NHM, Cromwell Road, London SW7 5BD, United Kingdom; 5 Departamento de Zoologia, Universidade Estadual Paulista – UNESP, IBB, Distrito de Rubião Júnior s/n, Botucatu, SP CEP 18618-970, Brazil

**Keywords:** America, Diaptominae, Diversity, Evolution, GIS, PAE, Richness

## Abstract

**Introduction:**

Diaptomid copepods are prevalent throughout continental waters of the Neotropics, yet little is known about their biogeography. In this study we investigate the main biogeographical patterns among the neotropical freshwater diaptomid copepods using Parsimony Analysis of Endemicity (PAE) based on species records within ecoregions. In addition, we assess potential environmental correlates and limits for species richness.

**Results:**

PAE was efficient in identifying general areas of endemism. Moreover, only ecoregion area showed a significant correlation with diaptomid species richness, although climatic factors were shown to provide possible upper limits to the species richness in a given ecoregion.

**Conclusion:**

The main patterns of endemism in neotropical freshwater diaptomid copepods are highly congruent with other freshwater taxa, suggesting a strong historical signal in determining the distribution of the family in the Neotropics.

## Introduction

Diaptomid copepods are among the main trophic links between primary producers and consumers in aquatic webs and represent the dominant family of Calanoida in inland waters of Europe, Asia, North America (NA), Africa, and the northern part of South America (SA) [1]. Their origin, diversification, taxonomy, and evolution are still poorly understood in the Neotropics - particularly in South America - and there have been few studies dealing with general aspects of the regional biogeography of the group (but see [2,3]). Although the origin of the order Calanoida is ancient, dating back at least from the Silurian period (439-416 MYA) [4], the initial colonization by diaptomids of continental waters from marine origins is hypothesized to have taken place in the northern supercontinent of Laurasia sometime after the break-up of Pangaea (around 160 MYA) [1]. The success of this colonization and subsequent diversification process is reflected by the fact that nearly half of the current 440 known species in the family are distributed in the Palearctic and Nearctic regions [5]. It has been assumed that the colonization of the Neotropics is probably much more recent, but no theory has yet received wide acceptance. According to the scenario hypothesized by Boxshall & Jaume [1], the presence of diaptomids at low altitudes in the northern and central parts of SA resulted from a late invasion from NA, occurring after the closure of the Panama gap in the Pliocene about 3 MYA. After invading from the North, the diaptomids would have spread rapidly, colonizing the interconnected lowland river systems. In this scenario the rapid colonization of multiple river basins would conceal biogeographical differences among regions, as well as a possible N-S gradient in species richness [6], given that the southern areas would have had less time to accumulate species. In fact, patterns that would be congruent with this scenario can be observed in freshwater cyclopoid copepods [7], but they are inconsistent with the remarkably high diversity of SA diaptomids. Alternatively, Suárez-Morales [2] and Suárez-Morales *et al*. [3] suggested the development of independent diaptomid faunas in Central/North America and in SA, resulting from their long isolation prior to the closing (in the Pliocene) of the Panama isthmus. Such a recent connection would help to explain the large differences in diversity between the SA diaptomid fauna and that of Central America (CA).

An understanding of diaptomid biogeography is still in its infancy. Most studies to date either include restricted geographical regions [3,8] or are based on panbiogeography [9,10], which has been heavily criticized for its excessive reliance on ancient vicariance (to the expense of alternative hypotheses) and its limitations in terms of reproducibility (see [11] for a recent discussion). Given the lack of comprehensive phylogenetic information on neotropical diaptomids, a valuable first approximation can be obtained through a Parsimony Analysis of Endemicity (PAE, [12]). One important recent criticism of PAE is use of biologically unrealistic limits to areas, such as geopolitical boundaries [13]. Another, criticize the inability of PAE to detect perfect vicariance or dispersal to explain biogeographical histories trough phylogenetic biogeography [14]. In contrast, one advantage of the study of freshwater fauna is that hydrological basins provide natural geographical boundaries between areas (e.g. [15]), thus improving considerably the explanatory power of the analyses. A detailed discussion for and against the use of PAE can be found at Morrone [16].

The goal of the present study is to provide a large-scale study of the biogeography of neotropical diaptomid species using the most comprehensive dataset on species occurrences compiled to date. Our specific goals are (1) to use PAE as a tool to provide a general overview of the biogeographical relationships among diaptomid copepod faunas of different neotropical ecoregions; (2) to map the geographical variation in diaptomid species richness in the Neotropics; (3) to test the role of climatic factors as potential correlates of diaptomid species diversity.

## Results

**Figure 1:**
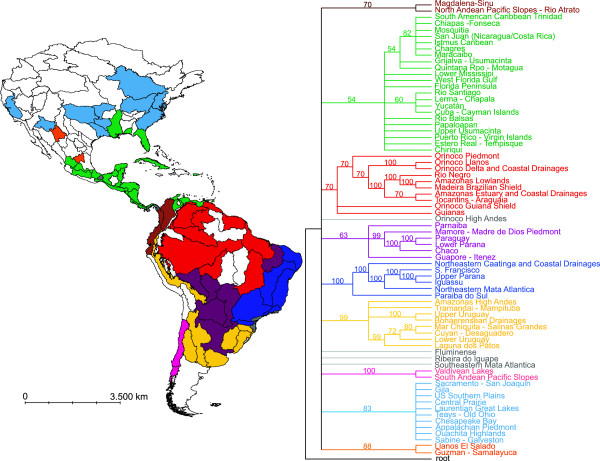
**The biogeographical relationships among ecoregions based on their diaptomid copepods, as inferred by PAE.** The biogeographical relationships among ecoregions based on their diaptomid copepods, as inferred by PAE. Ecoregion names follow Abell et al. [34]. Map colors correspond to the groupings indicated on the right. Uncolored regions of the map indicate ecoregions without any occurrence record of Diaptomidae.

The PAE results of the biogeographical relationships among ecoregions based on their diaptomid copepod fauna (Figure 1) revealed strong support for several groups of ecoregions, some of which showed interesting sub-structuring. A large cluster of ecoregions encompasses the higher Guiana shield and includes the ecoregions of the Guianas and the Orinoco river (Piedmont, Llanos, Guiana Shield, delta and coastal drainages), with the Amazon lowlands (Rio Negro, Amazonas Lowlands, Madeira Brazilian Shield, Tocantins-Araguaia, and Amazonas estuary and coastal drainages) nested within this cluster (Figure 1, in red). Another large cluster includes most of eastern Brazil (Paraíba do Sul, Northeastern Mata Atlantica, Iguassu, Upper Parana, S. Francisco, Northeastern Caatinga and coastal drainages) (Figure 1, dark blue). Some of the ecoregions found within the zone with relatively low precipitation located between the Amazon and the Atlantic Rainforest biomes (the “dry diagonal”) formed another cluster that included Parnaíba, Mamore Madre de Dios Piedmont, Paraguay, Lower Parana, Chaco, and Guapore Itenez (Figure 1, in purple). A fourth large cluster of ecoregions revealed an association of several ecoregions in the southern Neotropics (Laguna dos Patos, Lower Uruguay, Cuyan Desaguadero, Mar Chiquita Salinas Grandes, Bonaerensean Drainages, and Upper Uruguay), as well as some high elevation areas of the Andes (Amazonas High Andes and Tramandai Mampituba, Figure 1, in yellow). Other regions included some faunistically unique clusters, such as the Valdivian Lakes, the South Andean Pacific Slopes (Figure 1, in light pink), and the Magdalena Sinu/North Andean Pacific Slopes (Figure 1, in brown). The Central America ecoregions forms a large cluster (Figure 1, in green), which was divided into several small groups. Within these clusters, high support was found to group Caribbean ecoregions. Most North American ecoregions were faunistically distinct from Central American ecoregions (Figure 1, light blue).

**Figure 2:**
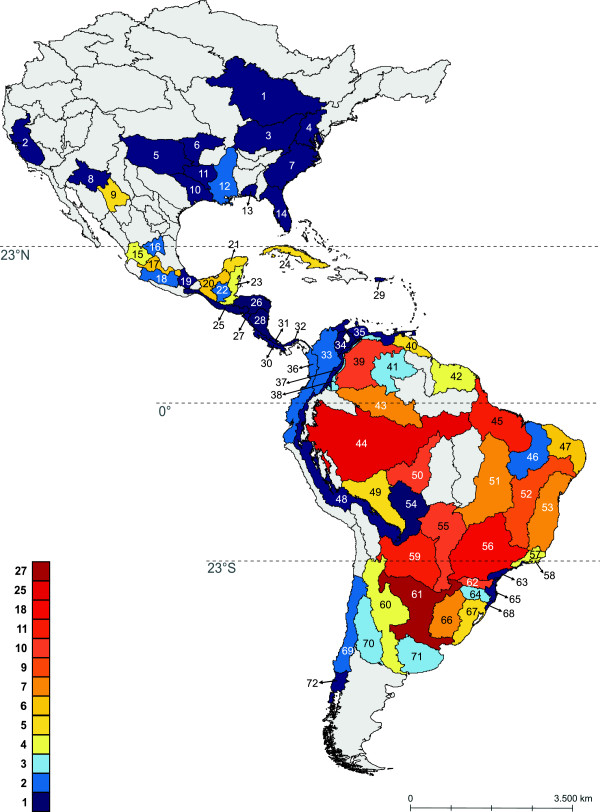
**Geographical distribution of species richness of neotropical freshwater diaptomidae across ecoregions.** Geographical distribution of species richness of neotropical freshwater Diaptomidae across ecoregions. Numbers correspond to ecoregion names indicated in Additional file 1: Table S1.

**Figure 3:**
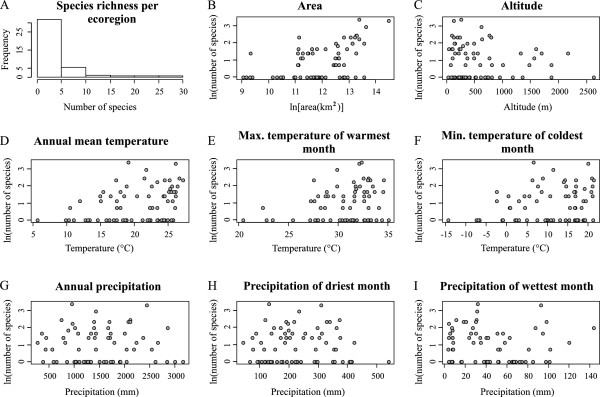
**Diaptomid species richness and candidate correlates. A**. Species richness per ecoregion. The remaining scatterplots indicate the relationship between species richness and candidate correlates, namely area **(B)**, altitude **(C)**, annual mean temperature **(D)**, maximum temperature of warmest month **(E)**, minimum temperature of the coldest month **(F)**, annual precipitation **(G)**, precipitation of driest month **(H)**, and precipitation of wettest month **(I)**. See text for details, and also Additional file 1: Table S2 for average climatic raw data.

Ecoregions varied considerably in their respective number of recorded species (Figure 2). However, such differences were not structured along a simple latitudinal gradient. Rather, most diaptomid species are found east of the Andes, particularly in the Amazon lowlands and in the Lower Paraná (Figure 2). Interestingly, of all tested candidate correlates, only the area of the ecoregion showed a significant association with diaptomid species richness (Table 1, F = 7.97, p = 2.5e-07, adjusted r^2^ = 0.44). This is intriguing, given the considerable climatic differences between ecoregions. The reason for this result becomes clear by inspecting the actual scatterplots of each variable against species richness (Figure 3). For instance, although ecoregions with low annual temperature were species-poor, higher temperatures include ecoregions with an increasingly large variation of species richness. In other words, although colder regions have consistently fewer species, warmer conditions include both low and high values of species richness. This suggests that, instead of a simple linear relationship between species richness and environmental variables, the climatic conditions found in a given ecoregion might only provide an “upper boundary” of the total species richness that can be found there. This inference was supported by our results of the BSS analyses for mean annual temperature (p = 0.002), maximum temperature during the warmest month (p = 0.06), and minimum temperature during the coldest month (p = 0.02), whereas altitude was only marginally significant (p = 0.09).

**Table 1:** Multiple regression analysis of potential correlates of neotropical freshwater diaptomid species richness

**Variable**	**Estimate**	**Std. error**	**t value**	**Pr (>|t|)**
log (area)	0.45	0.08	5.84	2.1e-07
Altitude	0.00	0.00	−0.60	0.55
Annual mean temperature	0.01	0.01	0.35	0.73
Maximum temperature of warmest month	0.00	0.01	−0.53	0.60
Minimum temperature of coldest month	0.01	0.01	0.70	0.49
Annual precipitation	0.00	0.00	−0.37	0.71
Precipitation of driest month	0.00	0.01	0.57	0.57
Precipitation of wettest month	0.00	0.00	−0.50	0.62

## Discussion

The present study represents the most comprehensive continental-scale biogeographical investigation of freshwater copepods to date and the first to use ecoregion divisions as biogeographical units. Our results provide valuable insight into the distribution patterns of the organisms at different geographic scales, building on the earlier works of Brandorff [17], Dussart [18] and Suárez-Morales *et al*. [3] for the Neotropics. In particular, ecoregions provided congruent physical limits to explain the copepod distributional patterns through the PAE, as previously demonstrated for teleosts [19]. The main ecoregions in terms of endemicity are Amazonas Lowland, Amazonas Estuary and Coastal Drainages, Lower Paraná, Chaco, Orinoco, Llanos, Paraguay, Iguassu, Madeira Brazilian Shield, Tocantins Araguaia, Northeastern Mata Atlantica, and Lower Uruguay. Some of these areas might represent diversity hotspots, and faunal differences between those regions should be taken into account in conservation initiatives.

The historical scenario elaborated by Boxshall & Jaume [1] assuming an extensive colonization and rapid speciation following the closure of the Panama gap in the Pliocene (about 3 MYA) would suggest that insufficient time had been available to allow biogeographical differentiation between regions, but our results do not support those predictions. In contrast, we found extensive differentiation between the diaptomid faunas of the ecoregions throughout the Neotropics. The high species richness of South American diaptomids is highly congruent with those observed for other freshwater taxa such as teleost fish (e.g. [20,21]). The species richness of diaptomids was positively correlated with ecoregion area, and in this taxon the highest values were found in the largest hydrographic basins (the Amazonas and Paraná rivers), a pattern shared also with fish [19]. The high diversity and endemicity of diaptomids in SA suggest an ancient occupation of this part of the neotropical region, prior to the Pliocene. The lowlands of Central and part of mainland Central America were subject to repeated marine transgressions, such that the current diaptomid fauna of the region was established during the Holocene, 8,000 years ago, by successive local colonization and extinction events [2]. We infer that diaptomid copepods were distributed in SA over period of time long enough for the group to have undergone major diversification but also to remain isolated from Central America. The weak influence of the SA diaptomid fauna in Central America and Mexico, being represented only by the presence of *Prionodiaptomus colombiensis*[3,22], together with the biogeographic affinity between the northernmost sector of South America and Central America (Figure 1) provides additional support for this inference. This interpretation is also supported by the species richness patterns of teleost fish and freshwater harpacticoid copepods. In both taxa the representation of SA species in CA is weak and attributed to recent, post-Pleistocene invasions [20,23].

Central American Diaptomids are also known to occur in the Nearctic region, and may possibly be the origin of the main colonizing populations. Some species are also shared with the Palaearctic region [2,3]. This suggests a weak influence of SA diaptomids, with exception of only two genera. The same occurs with the order Harpacticoida from high altitudes [23] as well as for other aquatic taxa, such as fish [20], a pattern attributed to desiccation processes in these regions [20]. The opposite pattern has been revealed for cyclopoid copepods, with a strong SA influence in Central America [7], emphasizing that the appearance of these organisms is regarded as relatively recent [4], from the Neogene (15 MYA). Suárez-Morales & Reid [24] suggested at least five biogeographic groups for American diaptomids, which are supported by the present study using PAE. The actual number of diaptomid species might be underestimated in some genera that presumably radiated in Central America, such as *Leptodiaptomus* and *Mastigodiaptomus*[3]. New taxonomically-oriented efforts and biogeographic data will reveal more detailed local patterns in this subregion. Nevertheless, it is likely that the general trend showing relatively lower diaptomid diversity with respect to South America will prevail.

In general, Central American ecoregions have smaller areas than the SA counterparts - a difference that could explain the lower species richness in CA and also in SA regions with restricted areas along the coastal basins of the Atlantic Ocean and Brazil. These ecoregions also have low teleost species richness [19]. The higher species richness of diaptomids found in larger ecoregions is in accord with the results of previous analyses estimating the relationships between species number and area for planktonic crustaceans [25]. Highly diverse areas might be characterized as biodiversity hotspots, as suggested for other groups of organisms [19], because of the increasing anthropic activities and potential disturbance of these sites. Such information should also be used to inform policies and conservation actions at different levels [26].

The Amazon ecoregion is impacted by an intense and increasing deforestation [27] and the construction of dams for hydroelectric energy along the main rivers (i.e. Madeira, Tapajós, Teles-Pires, Araguaia, Tocantins and Xingu) has had an additional impact. The reservoirs bring about a permanent change in surrounding habitats, such as lakes and marshes, and some studies suggest that copepod diversity is lower in reservoirs compared to lotic stretches of rivers [28]. The Paraná River basin is extensively dammed and is also the location of the largest cities in SA, which contribute to raised water pollution and eutrophication. Only a few tolerant species thrive under such conditions and they are found in ecoregions that include the Paraná River basin. Some endemic species occur only in smaller ecoregions, and this highlights an important issue when considering species conservation: populations within smaller regions may be more susceptible to extinction brought about by various factors, including anthropogenic impact.

Little is known about the ecological drivers of species richness in freshwater copepods at larger scales, although climate has been recognized as an important variable impacting most components of regional fauna and flora [29]. Variables such as lake area, conductivity, pH, dissolved inorganic carbon, and chlorophyll can all show correlations with copepod abundance (e.g. [25,30]). In SA there are several polythermic species, which are likely to tolerate wide variation in temperature [17]. In addition, the progressive increase of temperature driven by global warming has been implicated as responsible for changes in the centers of distribution of some crustacean species at higher latitudes [31]. Our results suggest that a combination of climatic factors such as low precipitation and temperature, might determine an upper limit to the number of species that can be harbored within a given ecoregion, but more moderate climatic conditions within a particular geographical area do not necessarily lead to higher species richness. The highest levels of diaptomid diversity were linked with the largest ecoregions, supporting the inference that suitable conditions for the maintenance of high species richness were more likely to be stable in larger ecoregions. Based on the results of the BSS correlations undertaken in this study, we infer that minimum temperatures are a boundary condition for species dwelling in high temperature ecoregions, because of the high thermal variation in colder places. However, these conclusions should be interpreted with care, given that sampling effort has not been homogeneous throughout the Neotropics and therefore could have affected some of the observed patterns and/or masked potential correlates of species richness.

## Conclusions

Despite the high environmental diversity and the complex biogeographic history of the Neotropics, diaptomid copepods show relatively well-defined patterns that are informative their underlying diversification processes. In particular, the relationships between areas of endemism and the high diversity in SA both support a history of diversification among copepods that preceded considerably the connection between the Nearctic and the Neotropics in the Pliocene. Finally, climatic conditions might provide boundaries to the level of diaptomid species richness that can be attained in a given ecoregion.

## Material and methods

Records of freshwater diaptomid species occurrences were compiled from an extensive survey of the literature (Additional file 1: Appendix S1), as well as from new and recent data [32]. The resulting dataset involves 98 species recorded in the Neotropics, although some are also found in the Nearctic region. This number of species differs from previous accounts (116 species of [3]; 82 from [5]). Each species was then scored as either present or absent in each of 72 ecoregions delimited according to the FEOW (Freshwater Ecoregions of the World, [33]) classification ([34], available at http://www.feow.org) (Additional file 1: Table S1). The resulting incidence matrix was investigated using Parsimony Analysis of Endemicity (PAE). Parsimony analyses were carried out using PAUP* v 4.0b10 [35]. Heuristic methods consisted in 1000 random taxon addition searches followed by tree-bisection reconnection. The 100 best trees from each replicate were retained and the final tree was represented as a majority-rule consensus among all replicates. Data of occurrence of Diaptomids used in PAE are show in Additional file 1: Table S3.

Drivers of diaptomid species richness were investigated by multiple regression analysis of the number of species in each ecoregion against potential correlates, namely altitude, area, annual mean temperature, maximum temperature of warmest month, minimum temperature of coldest month, annual precipitation, precipitation of wettest month, and precipitation of driest month. Estimates of these variables were obtained from WorldClim v 1.4 [36] by generating 5000 random coordinates in each ecoregion, extracting their respective values at a resolution of 2.5’ using ArcGIS v 9.3 [37], and calculating their averages. Finally, we tested whether diaptomid species richness is constrained by environmental conditions by using the Boundary Sum of Squares (BSS) test implemented in EcoSim v 7.0 [38], which is applied as follows. First, the ranges of the observed richness and environmental variables across all ecoregions are used to delimit a rectangular region in bivariate space. A lower right triangle is created by connecting the data points (min x, min y), (max x, max y), and (max x, min y), and the average distance of the points falling outside the lower right triangular from the boundary is calculated. If this value is unusually small, the points are concentrated near that boundary. Statistical significance is obtained by random resampling (1000 times) of the original dataset. The entire procedure was repeated for the following variables: annual mean temperature, maximum temperature during the warmest month, and minimum temperature during the coldest month. The effect of altitude was tested using a similar approach, except that a lower left triangle was used by connecting the data points (min x, min y), (max x, min y), and (max x, max y).

### Availability of supporting data

The data sets supporting the results of this article (Additional file 1: Tables S1 and S2) are available as online Additional file 1.

## Competing interests

The authors declare that they have no competing interests.

## Authors’ contributions

GPN, DP, MRP and AD collected the data, led the writing and conceived the idea, and analyzed the data; ESM, GAB, MGN and CEFR led the writing. All authors read and approved the final manuscript.

## Authors’ information

Gilmar Perbiche Neves is a post-doctoral researcher of University of São Paulo (USP, Brazil). He works on freshwater copepods, investigating several areas as biogeography, ecology, taxonomy, evolution, molecular and morphological phylogeny, etc., and his academic interests center on understanding the evolution of freshwater copepods.

## Supplementary Material

Additional file 1 References compiled to generate the dataset used in the present study. **Table S1.** Ecoregions included in the present study. Numbers correspond to ecoregions indicated in Figure 2. **Table S2.** Average climatic data associated with each ecoregion. **Table S3.** Occurrence data on Neotropical diaptomids used for Parsimony Analysis of Endemicity. See **Table S4.** for the names of each ecoregion. **Table S4.** Column names for the data matrix used for Parsimony Analysis of Endemicity indicated in **Table S3.** Click here for file
