# Supplementary material for: Historical biogeography of the neotropical Diaptomidae (Crustacea: Copepoda)
Source: Front Zool. 2014 May 1;11:36. doi: 10.1186/1742-9994-11-36 (PMC4108091; doi:10.1186/1742-9994-11-36)
Supplement: Additional file 1 — References compiled to generate the dataset used in the present study. Table S1. Ecoregions included in the present study. Numbers correspond to ecoregions indicated in Figure 2. Table S2. Average climatic data associated with each ecoregion. Table S3. Occurrence data on Neotropical diaptomids used for Parsimony Analysis of Endemicity. See Table S4. for the names of each ecoregion. Table S4. Column names for the data matrix used for Parsimony Analysis of Endemicity indicated in Table S3. [file 1742-9994-11-36-S1.pdf]

Online Supplementary Material

Appendix S1. References compiled to generate the dataset used in the present study:

Almeida VLS, Melão MG, Moura NA: **Plankton diversity and limnological characterization in two shallow tropical urban reservoirs of Pernambuco State, Brazil**. *An Acad Bras Ciênc* 2012, **84**(2): 537-550.

Bohrer MBC, Araújo PB: **Subclasse Copepoda (espécies de águas continentais)**. In: *Os Crustáceos do Rio Grande do Sul*. Edited by Buckup L, Bond-Buckup G. Porto Alegre: Ed. Universidade/UFRGS; 1999: 92-105.

Bonecker CC, Nagae MY, Bletller MCM, Velho LFM, Lansac-Tôha FA: **Zooplankton biomass in tropical reservoirs in southern Brazil**. *Hydrobiologia* 2007, **579**: 115-123.

Bonecker CC, Bonecker SLC, Bozelli RL, Lansac- Tôha FA, Velho LFM: **Zooplankton composition under the influence of liquid wastes from a pulp mill in Middle Doce River (Belo Oriente, MG, Brazil)**. *Arq Biol Tecnol* 199639: 893-901.

Bonecker CC, Lansac-Tôha FA, Velho LFM, Rossa DC: **The temporal distribution patterns of copepods in Corumbá Reservoir, State of Goiás, Brazil**. *Hydrobiologia* 2001, **453/454**: 375-384.

Bozelli RL, Thomaz SM, Roland F, Esteves FA: **Variações nictemerais e sazonais de alguns fatores limnológicos na represa municipal de São José do Rio Preto, São Paulo**. *Acta Limnol Brasil* 1992, **6**: 53-66.

Brandorff GO: **Ein Beitrag zur Calanidenfauna (Crustacea, Copepoda) des Amazonasgebietes, mit einem Überblick über die Diaptomiden (Crustacea, Copepoda) Südamerikas** . *M.Sc. Thesis*. Universität Kiel; 1972.

Brandorff GO: **Distribution of some Calanoida (Crustacea: Copepoda) from the Yucatán Peninsula, Belize and Guatemala.** *Rev Biol Trop* 2012, **60**(1): 187-202.

Brandorff GO, Koste W, Smirnov NN: **The composition and structure of Rotiferan and Crustacean Communities of the Lower Rio Nhamundá, Amazonas, Brazil**. *Stud Neotrop Fauna E* 1982, **17**: 69-121.

Brandorff GO: **Neue freilebende calanoide Copepoden (Crustacea) aus den Amazonasgebiet**. *Amazoniana* 1973, **4**: 205-218.

Brehm V: **Über die Süsswassserfauna von Uruguay**. *Arch Hydrobiol* 1935, **28**: 295-309.

Brehm V: **Nachträgliche Notizen zür Süßwasserfauna von Uruguay**. *Zool Anz* 1938, **7**(1): 26-31.

Brehm V: **Bemerkungen zur einigen Kopepoden Südamerikas**. *SOeAW* 1959, **1**(168): 497-521.

Brehm V: **Bericht über eine unvollendet gebliebene Untersuchung der Argentinischen Kopepodenfauna**. *SOeAW* 1965, **1**(174): 1- 15.

Brian A: **Di alcuni copepodi d`acqua dolce dell'Argentina**. *Mem Soc Entomol Ital* 1926, **4**: 177-200.

Brito SL, Maia-Barbosa PM, Pinto-Coelho RM: **Zooplankton as an indicator of trophic conditions in two large reservoirs in Brazil.** *Lakes Reserv Res Manage* 2011, 16: 253-264.

Campos H, Steffen W, Agüero G, Parra O, Zúñiga L: **Limnological studies in lake Villarrica: Morphometry, physics, chemistry and primary productivity**. *Arch Hydrobiol* 1983, **71**: 37-67.

Cardoso LS, Marques DM: **The Influence of Hydrodynamics on the Spatial and Temporal Variation of Phytoplankton Pigments in a Large, Sub-Tropical Coastal Lake (Brazil)**. *Braz Arch Biol Tech* 2004, **47**(4): 587-600.

Choueri RB, Bonecker CC, Dias JD: **Spatial and temporal density variation of microcrustacean assemblages in different systems of the upper Paraná river floodplain (PR/MS-Brazil)**. *Acta Sci Biol* 2005, **27**(3): 243-250.

Cicchino G, Zoppi de Roa E, Montiel E: ***Notodiaptomus henseni*** **Dahl (Crustacea-Copepoda): un problema de sinonimia en el zooplancton de Venezuela**. *Acta Biol Venez* 1989, **12**: 98-105.

Cipólli MN: **Morfologia externa das fases de desenvolvimento de *Diaptomus corderoi* Wright, 1936 (Crustacea, Copepoda, Calanoidea).** *Biol Zool e Biol Mar* 1973**, 30**: 567-612.

Correia de Melo NFA, Paiva RS, Silva MMR: **Considerações ecológicas sobre o zooplâncton do lago Bolonha, Belém, Pará, Brasil**. *Bol Mus Para Emílio Goeldi* 2006, **1**(1): 115-125.

Dabés MBGS: **Composição e descrição do zooplâncton de cinco lagoas marginais do Rio São Francisco, Pirapora, Três Marias, Minas Gerais – Brasil.** *Rev Bras Biol* 1995, **55**(4): 831-845.

Daday E: **Untersuchungen über die Süsswassermikrofauna Paraguays, VI**. **Copepoda.** *Zoologica Stuttgart* 1905, **18**(44): 131-153 + pls. 8,9.

De los Ríos-Escalante P, Hauenstein E, Romero-Mieres M*:* **Microcrustacean assemblages composition and environmental variables in lakes and ponds of the Andean region – South of Chile (37-39° S)**. *Braz J Biol* 2011, **71**(2): 353-358.

Dodson SI, Silva-Briano M: **Crustacean zooplankton species richness and associations in reservoirs and ponds of Aguas Calientes, México**. *Hydrobiologia* 1996, **325**: 163-172.

Dussart BH, Fernando CH: **Tropical freshwater Copepoda from Papua, New Guinea, Burma, and Costa Rica, including a new species of *Mesocyclops* from Burma**. *Can J Zool* **63**: 202-206.

Dussart BH, Frutos SM: **Sur quelques Copépodes d´Argentine: 2. Copépodes du Paraná Medio**. *Rev Hydrobiol Trop* 1986, **19**(3-4): 241-262.

Dussart BH: **Sur Quelques Copépodes d'Amérique du Sud.** IV. *Rev Bras Biol* 1984, **44**(3) : 255-265.

Dussart BH: **Sur quelques copépodes d'Amérique du Sud, V. Diaptomidae**. *Arch Hydrobiol* 1985, **103**: 201-215.

Elías-Gutiérrez M, Suárez-Moráles E, Sarma SSS: **Freshwater zooplankton diversity in the Neotropics: the case of Mexico**. *Verh Int Verein Limnol* 2001, **27**: 1–5.

Eskinazi-Sant'Anna E, Menezes R, Costa IS, Panosso RF, Araújo MF, Attayde JL: **Composição da comunidade zooplanctônica em reservatórios eutróficos do semi-árido do Rio Grande do Norte**. *Oecol Bras* 2007 **11**(3): 410-421.

Espíndola ELG, Matsumura-Tundisi T, Rietzler AC, Tundisi JG: **Spatial heterogeneity of the Tucuruí Reservoir (state of Pará, Amazonia, Brasil) and the distribution of zooplanktonic species**. *Rev Bras Biol* 2000, **60**: 179-194.

Gavíria S, Aranguren N: **Free-living species of the Copepoda (Arthropoda, Crustacea) subclass of the Colombian continental waters**. *Biota Colomb* 2007, **8**: 53-68.

Gouvêa EP: **Estágios do desenvolvimento naupliar de *Notodiaptomus conifer* (Sars, 1901) (Copepoda, Calanoida)**. *Ciênc Cult* 1980, **32**: 1047-1059.

Granados-Ramírez J, Suárez-Morales E: **A new *Hesperodiaptomus* Light (Copepoda, Calanoida, Diaptomidae) from Mexico with comments on the distribution of the genus**. *J Plankton Res* 2003, **25**: 1383-1395.

Gutierrez-Aguirre M, Cervantes-Martinez A: **Diversity of freshwater copepods (Maxillopoda: Copepoda: Calanoida, Cyclopoida) from Chiapas, Mexico with a description of *Mastigodiaptomus suarezmoralesi* sp. nov**. *J Nat Hist* 2013, **47**: 479–498.

Gutiérrez-Aguirre MA, Suárez-Morales E: **Diversity and distribution of freshwater copepods (Crustacea) in southeastern Mexico**. *Biodivers Conserv* 2001, **10**: 659–672.

Gutiérrez-Aguirre MA, Cervantes-Martínez A, Elías-Gutiérrez M: **An Example of How Barcodes Can Clarify Cryptic Species: The Case of the Calanoid Copepod *Mastigodiaptomus albuquerquensis* (Herrick)**. *Plos One*, 2014, **9**(1): 1-11.

José de Paggi S: **First observations on longitudinal succession of zooplankton in the main course of the Paraná River between Santa Fé and Buenos Aires harbour**. *Stud Neotrop Fauna* 1978, **13**: 143-156.

Kiefer F: **Freilebende Ruderfusskrebse (Crustacea Copepoda). I. Calanoida und Cyclopoida**. In: *Ergebnisse der Deutschen Limnologischen Venezuela-Expedition 1952.* 1956, **1**: 233-268.

Lansac-Tôha FA, Bonecker CC, Velho LFM, Lima AF: **Comunidade zooplanctônica**. In *A planície de inundação do alto rio Paraná: aspectos físicos, químicos, biológicos e socioeconômico.* Edited by Vazzoler AEAM, Agostinho AA, Hahn NS. Maringá: Editora da Universidade Estadual de Maringá; 1997: 117-155.

Lansac-Tôha FA, Bonecker CC, Velho LFM, Simões NR, Dias JD, Alves GM, Takahashi EM: **Biodiversity of zooplankton communities in the Upper Paraná River floodplain: interannual variation from long-term studies**. *Braz J Biol* 2009, **69**(2): 539-549. Doi: 10.1590/S1519-69842009000300009

Loftler H: **Zur Systematik and Okologie der chilenische Susswasserentomostraken**. *Beitr neotrop fauna* 1961, **2**: 143-222 .

Lowndes AG: **Reports of an expeditions to Brazil and Paraguay in 1926-7 supported by the Trustees of the Percy Sladen Memorial Fund and the executive Committee of the Carniegie Trust of Scotland**. *Biol J Linn Soc* 1934, **39**: 83-131. Doi: 10.1111/j.1096-3642.1934.tb00260.x

Marsh CD: **Report on fresh-water copepods from Panama, with descriptions of new species**. *Smithsonian Misc Collect* 1913, **61**: 1-31 + pls. 1-5.

Matsumura-Tundisi T, Tundisi JG: **Calanoida (Copepoda) species composition changes in the reservoirs of São Paulo State (Brazil) in the last twenty years**. *Hydrobiologia* 2003, **504**: 215-222. Doi: 10.1023/B:HYDR.0000008521.43711.35

Matsumura-Tundisi T: **Latitudinal distribution of Calanoida copepods in freshwater aquatic systems of Brazil**. *Rev Bras Biol* 1986, **46**(3): 527–553.

Matsumura-Tundisi T: **Rectification of description of *Notodiaptomus spinuliferus* Dussart and Matsumura-Tundisi, 1986**. *Brazilian J Biol* 2008, **68**(3): 683-684. Doi: 10.1590/S1519-69842008000300029

Melo Jr. MM, Almeida VLS, Paranaguá MN, Moura NA: **Crustáceos planctônicos de um reservatório oligotrófico do Nordeste do Brasil.** *Rev Bras Zoociênc* 2007, **9**(1): 19-30.

Montiel-Martínez A, Ciros-Pérez J, Ortega-Mayagoitia E, Elías-Gutiérrez M: **Morphological, ecological, reproductive and molecular evidence for *Leptodiaptomus garciai* (Osorio-Tafall 1942) as a valid endemic species**. *J Plankton Res* 2008, **30**(10): 1079-1093. Doi: 10.1093/plankt/fbn067.

Montú M, Gloeden IM: **Atlas dos Cladocera e Copepoda (Crustacea) do estuário da lagoa dos Patos (Rio Grande, Brasil)**. *Nerítica* 1986, **1**: 1-134.

Nogueira MG: **Zooplankton composition, dominance and abundance as indicators of environmental compartmentalization in Jurumirim Reservoir (Paranapanema River), São Paulo, Brazil**. *Hydrobiologia* 2001, **455**: 1-18. Doi: 10.1023/A:1011946708757

Nogueira MG, Reis-Oliveira PC, Britto YT: **Zooplankton assemblages (Copepoda and Cladocera) in a cascade of reservoirs of a large tropical river (SE Brazil)**. *Limnetica* 2008, **27**(1): 151-170.

Paggi JC: **Clarification of the taxonomic status of *Notodiaptomus anisitsi* (Daday, 1905) and related species, with description of a new species from Argentina (Crustacea: Copepoda: Diaptomidae)**. *Hydrobiologia* 2001, **453/454**: 549-564. Doi: 10.1023/A:1013199713987

Paggi JC: **Redescription and re-evaluation of the taxonomic status of the Neotropical copepod *Diaptomus falcifer* Daday, 1905 (Calanoida: Diaptomidae).** *Stud Neotrop Fauna*, 2006, **41**(1): 67-78. Doi: 10.1080/01650520500233687

Paggi JC: **A new species of the rare genus *Idiodiaptomus* Kiefer, 1936 (Copepoda, Calanoida, Diaptomidae) from northeastern Argentina**. *Crustaceana Monographs* 2011, **16**: 1570-7024.

Paggi JC, José de Paggi S: **Zooplankton de ambientes lóticos e lênticas do rio Paraná médio**. *Acta Limnol Bras* 1990, **3**: 685-719.

Perbiche-Neves G, Serafim-Júnior M, Ghidini AR, Brito L: **Spatial and temporal distribution of Copepoda (Cyclopoida and Calanoida) of an eutrophic reservoir in the basin of upper Iguaçu River, Paraná, Brazil.** *Acta Limnol Bras* 2007, **19**(4): 393-406.

Perbiche-Neves G, Previattelli D, Nogueira MG: Record of *Argyrodiaptomus bergi* (Crustacea: Copepoda: Calanoida) after 36 years and first record in Brazil. *Zoologia* 2011, 28(5): 551-557. Doi: 10.1590/S1984-46702011000500001

Perbiche-Neves G, Nogueira MG: **Reservoir design and operation: effects on aquatic biota – a case study of planktonic copepods.** *Hydrobiologia* 2013, **707**: 187–198. doi:10.1007/S10750-012-1425-1

Previatelli D, Santos-Silva EN: **A new *Argyrodiaptomus* (Copepoda: Calanoida: Diaptomidae) from the southwestern Brazilian Amazon.** *Zootaxa,* 2007, **1518**: 1-29.

Previattelli D, Perbiche-Neves G, Santos-Silva EN: **New Diaptomidae records (Crustacea: Copepoda: Calanoida: Diaptomidae) in the Neotropical region**. *CheckList,* 2013, **9**(4): 700–713.

Reid JW, Hribar LJ: **Records of some Copepoda (Crustacea) from the Florida Keys.** *Proc Acad Nat Sci Philadelphia* 2006, **155**: 1-7.

Reid JW, Pinto-Coelho RM:. **Planktonic Copepoda of Furnas Reservoir: initial survey of species (1993) and review of literature**. In *Ecology and Human Impact on Lakes and Reservoirs in Minas Gerais with Special Reference to Future Development and Management Strategies*. Edited by Pinto-Coelho RM, Giani E, Von Sperling E. Belo Horizonte: SEGRA;. 1994: 93-114.

Reid JW, Turner PN: **Planktonic Rotifera, Copepoda and Cladocera from lagos Açú and Viana, State of Maranhão, Brazil**. *Rev Bras Biol* 1988, **48**: 485-495.

Reid JW: **Calanoid copepods (Diaptomidae) from coastal lakes, State of Rio de Janeiro, Brasil**. *P Biol Soc Wash* 1985, **98**: 574-590.

Reid JW: ***Scolodiaptomus*, a new genus proposed for *Diaptomus* (*sensu lato*) *corderoi* Wright, and description of *Notodiaptomus brandorffi*, new species (Copepoda: Calanoida), from Brazil.** *J Crust Biol* 1987, **72**: 64-379. Doi: 10.2307/1548616

Reid JW, Moreno IH: **The Copepoda (Crustacea) of the southern Pantanal, Brasil**. *Acta Limnol Bras* 1990, **3**: 721-739.

Reid JW: ***Argyrodiaptomus nhumirim*, a new species, and *Austrinodiaptomus kleerekoperi*, a new genus and species, with redescription of *Argyrodiaptomus macrochaetus* Brehm, new rank, from Brazil (Crustacea: Copepoda: Diaptomidae).** *P Biol Soc Wash* 1997, **110**(4): 581-600.

Reid JW: ***Arctodiaptomus dorsalis* (Marsh): A case history of copepod dispersal**. *Banisteria* 2007, **30**: 3-18.

Ringuelet RA: **Primeros datos ecológicos sobre copépodos dulciacuícolas de la República Argentina**. *Physis Buenos Aires*, 1958, **21**(60): 14-31.

Ringuelet RA: **Rasgos faunísticos de las reservas naturales de la Provincia de Buenos Aires.** *Physis Buenos Aires* 1962, **23**(64): 83-92.

Rocha O, Rietzler AC, Espíndola, Matsumura-Tundisi T, Dumont HJ: **Diversity of fauna in sand dune lakes of Lençóis Maranhenses, Brazil, I: the zooplankton community**. *An Acad Bras Ciênc* 1998, **70**: 793-795.

Santos-Silva EM, Robertson BA: **A new species of *Rhacodiaptomus* Kiefer, 1936 from the Brazilian Amazon (Crustacea: Copepoda: Calanoida)**. *Acta Amaz* 1993, **23**: 95-105.

Santos-Silva EN: **Calanoid of the families Diaptomidae, Pseudodiaptomidae, and Centropagidae from Brazil**. *Biol G Exp* 2008, **8**(1): 3-67.

Santos-Silva EM, Robertson BA, Reid JW, Hardy ER: **Atlas de copépodos planctônicos, Calanoida e Cyclopoida (Crustacea), da Amazônia Brasileira. I. Represa de Curuá-Una, Pará**. *Rev Bras Zool* 1989, **6**: 725-758.

Saunders JF, Lewis Jr M: **Zooplankton abundance in the Caura River, Venezuela**. *Biotropica* 1988, 20: 206–214.

Sendacz S, Kubo E: **Copepoda (Calanoida e Cyclopoida) de reservatórios do Estado de São Paulo**. *B Inst Pesca* 1982, **9**: 51-89.

Sendacz S: **Zooplankton studies of floodplain lakes of the Upper Paraná River, São Paulo State, Brazil**. *Verh Int Ver Limnol* 1997, **26**: 621-627.

Sendacz S: **Planktonic Copepoda of the Upper Paraná River Floodplain lakes (São Paulo and Mato Grosso do Sul, Brazil)**. *Hydrobiologia* 2001, **453/454**: 367–374.

Sendacz S, Kubo E, Cestarolli MA: **Limnologia dos reservatórios do Estado de São Paulo, Brasil. VIII. Zooplâncton.** *B Inst Pesca* 1985, **12**: 187-207.

Silva WM, Roche KF, Vicente F, Delben AST: **First Record of the Peritrich *Trichodina diaptomi* Basson and Van As, 1991 (Protozoa: Ciliophora) on a South American Calanoid *Notodiaptomus deitersi* (Poppe, 1890) (Crustacea: Copepoda).** *J Eukaryot Microbiol* 2009, **56**(4): 385.

Silva-Briano M, Suárez-Morales E: **Illustrated record of the freshwater copepod *Leptodiaptomus dodsoni* (Calanoida, Diaptomidae) from central Mexico with comments on the distribution of the genus.** *Biota Neotrop* 2010, **10**(2): 1-6.

Simões NR, Sonoda SL: **Estrutura da assembleia de microcrustáceos (Cladocera e Copepoda) em um reservatório do semi-árido Neotropical, Barragem de Pedra, Estado da Bahia, Brasil**. *Acta Sci Biol* 2009, **31**(1): 89-95.

Simões, NR, Ribeiro SMMS, Sonoda SL: **Diversity and structure of microcrustacean assemblages (Cladocera and Copepoda) and limnological variability in perennial and intermittent pools in a semi-arid region, Bahia, Brazil.** *Iheringia, Sér Zool* 2011, **101**(4): 317-324.

Sipaúba-Tavares LH, Bachion MA, Braga FMS: **Effects of food quality on growth and biochemical composition of a calanoid copepod, *Argyrodiaptomus furcatus*, and its importance as a natural food source for larvae of two tropical fishes.** *Hydrobiologia* 2001, **453/454:** 393–401.

Smith K, Fernando CH: **The freshwater calanoid and cyclopoid copepod Crustacea of Cuba**. *Can J Zool* 1978, **56**: 2015-2023.

Soto D, Zuñiga LR: **Zooplankton assemblages of Chilean temperate lakes: A comparison with North American counterparts**. *Rev Chil Hist Nat* 1991, **64**: 569–581.

Spandl H: **Das Zooplankton des Paranagua-Sees**. *SOeAW* 1926, **76**: 101-105.

Starling FLRM: **Comparative study of the zooplankton composition of six lacustrine ecosystems in central Brazil during the dry season**. *Braz J Biol* 2000, **60**(1): 101-111.

Suárez-Morales E, Elías-Gutiérrez M: **On the taxonomical status of *Arctodiaptomus dampfi* Brehm (Crustacea: Copepoda: Diaptomidae) with comments on *A. dorsalis* (Marsh)**. *J Limnol* 2001, **60**: 11-18.

Suárez-Morales E: **Historical biogeography and distribution of the freshwater calanoid copepods (Crustacea: Copepoda) of the Yucatan Peninsula, Mexico**. *J Biogeogr* 2003, **30**(12): 1851–1859.

Suárez-Morales E, Silva-Briano M, Elías-Gutiérrez M: **Redescription and taxonomic validity of *Leptodiaptomus cuauhtemoci* (Osorio-Tafall, 1941) (Copepoda, Calanoida), with notes on its known distribution**. *J Limnol* 2000, **59**(1): 5-14.

Suárez-Morales E, Reid JW, Elías-Gutiérrez M: **Diversity and Distributional Patterns of Neotropical Freshwater Copepods (Calanoida: Diaptomidae)**. *Int Rev Hydrobiol* 2005, **90**(1): 71–83.

Suárez-Morales E, Gutiérrez-Aguirre M, Walsh EJ: **Freshwater Copepoda (Crustacea) from the Chihuahuan desert with comments on biogeography**. *Southwest nat* 2010, **55**(4): 525–531.

Thomasson K: **Studies on South American freshwater plankton. 3. Plankton from Tierra del Fuego and Valdivia.** *Acta Horti Gothob* 1955, **19**: 193-225.

Tundisi JG, Matsumura-Tundisi T: **Plankton diversity in a warm monomictic lake (Dom Helvécio, Minas Gerais) and a polymictic reservoir (Barra Bonita): A comparative analysis of the intermediate disturbance hypothesis**. *An Acad Bras Ciênc* 1994, **6**(1, I): 15-28.

Twombly S, Lewis Jr WM: **Zooplankton abundance and species composition in Laguna Orsinera, a Venezuelan floodplain lake.** *Arch Hydrobiol* 1987, **79**: 87-107.

Vásquez EZ, Sánchez L: **Variación estacional del plancton en dos sectores del río Orinoco y una laguna de inundación adyacente**. *Mem Soc Cienc Nat La Salle* 1984, **121**: 11–33.

Villalobos L, Parra O, Grandjean O, Jaque E, Woelfl O, Campos H: **River basin and limnological study in five humic lakes of the Chiloé Island**. *Rev Chil Hist Nat* 2003, **76**: 10-15.

Wright S: **A revision of the South American species of *Diaptomus***. *T Am Microsc Soc*, 1927, **46** (2): 73–121.

Wright S: **Preliminary report on six new species of *Diaptomus* from Brazil**. *An Acad Bras Ciênc* 1936, **8**: 79–85 + pls. 1, 2.

Wright S: **A review of the *Diaptomus bergi* group, with descriptions of two new species**. *T Am Microsc Soc*, 1938, **57**: 297-315.

Wright S: **Algunas especies del género "*Diaptomus*" hallados en la República Argentina**. *Physis Buenos Aires*, 1939, **17**: 645-649.

Zoppi de Roa E: **Seasonal variations of copepods from a flooding savanna of Venezuela.** *Verh Int Ver Limnol* 1994, **25**: 1383-1388.

Zuñiga LR, Araya JM: **Estructura y distribución, durante un periodo otoñal del zooplancton en el embalse Rapel.** *Anales Mus Hist Nat Valpso* 1982, **15**: 45-57.

Zúñiga LR: **Taxocenosis de entomostracos limnéticos de lagos del norte de la Patagonia.** *Anales Mus Hist Nat Valpso* 1988, **19**: 5-14.

Table S1. Ecoregions included in the present study. Numbers correspond to ecoregions indicated in figure 2.

n°	Ecoregion
1	Laurentian Great Lakes
2	Sacramento - San Joaquin
3	Teays - Old Ohio
4	Chesapeake Bay
5	US Southern Plains
6	Central Prairie
7	Appalachian Piedmont
8	Gila
9	Guzman - Samalayuca
10	Sabine - Galveston
11	Ouachita Highlands
12	Lower Mississipi
13	West Florida Gulf
14	Florida Peninsula
15	Rio Santiago
16	llanos El Salado
17	Lerma - Chapala
18	Rio Balsas
19	Papaloapan
20	Grijalva - Usumacinta
21	Yucatán
22	Upper Usumacinta
23	Quintana Roo - Motagua
24	Cuba - Cayman Islands
25	Chiapas - Fonseca
26	Mosquitia
27	Estero Real - Tempisque
28	San Juan (Nicaragua/Costa Rica)
29	Puerto Rico - Virgin Islands
30	Chiriqui
31	Istmus Caribean
32	Chagres
33	Magdalena - Sinu
34	Maracaibo
35	South American Caribbean Trinidad
36	North Andean Pacific Slopes - Rio Atrato
37	Orinoco High Andes
38	Orinoco Piedmont

39	Orinoco Llanos
40	Orinoco Delta and Coastal Drainages
41	Orinoco Guiana Shield
42	Guianas
43	Rio Negro
44	Amazonas Lowlands
45	Amazonas Estuary and Coastal Drainages
46	Parnaiba
47	Northeastern Caatinga and Coastal Drainages
48	Amazonas High Andes
49	Mamore - Madre de Dios Piedmont
50	Madeira Brazilian Shield
51	Tocantins - Araguaia
52	S. Francisco
53	Northeastern Mata Atlantica
54	Guapore - Itenez
55	Paraguay
56	Upper Parana
57	Paraiba do Sul
58	Fluminense
59	Chaco
60	Mar Chiquita - Salinas Grandes
61	Lower Parana
62	Iguassu
63	Ribeira do Iguape
64	Upper Uruguay
65	Southeastern Mata Atlantica
66	Lower Uruguay
67	Laguna dos Patos
68	Tramandai - Mampituba
69	South Andean Pacific Slopes
70	Cuyan - Desaguadero
71	Bonaerensean Drainages
72	Valdivean Lakes

Table S2. Average climatic data associated with each ecoregion.

Ecoregion	Species richness	Area (km^2)	Altitude (m)	Annual Mean Temperature (C^o)	Maximum Temperature of Warmest Month (C^o)	Minimum Temperature of Warmest Month (C^o)	Annual Precipitation (mm)	Precipitation of Wettest Month (mm)	Precipitation of Driest Month (mm)
Amazonas Estuary & Coastal Drainages	11	566418.50	95.05	26.61	33.01	20.92	2103.61	372.89	30.66
Amazonas High Andes	1	556331.30	2628.55	14.53	22.76	4.90	1249.22	191.32	39.17
Amazonas Lowlands	25	1911772.30	129.61	26.08	32.07	20.07	2438.49	308.38	92.65
Appalachian Piedmont	1	310693.50	143.73	15.80	31.82	-0.91	1207.59	134.73	74.46
Bonaerensean Drainages	3	265200.40	107.56	14.99	30.50	2.91	820.16	103.63	34.53
Central Prairie	1	158558.00	286.12	13.37	32.57	-6.70	1030.98	130.25	39.49
Chaco	11	589887.20	764.46	21.48	32.52	8.81	740.78	120.05	11.11
Chagres	1	11939.40	202.20	25.75	30.84	21.27	2841.53	413.01	40.94
Chesapeake Bay	1	142924.60	301.69	10.23	28.47	-7.43	1027.09	106.16	65.86
Chiapas Fonseca	1	90923.30	688.70	23.45	31.55	15.38	1723.59	348.07	6.33
Chiriqui	1	25615.00	608.73	23.38	30.38	17.19	3157.94	538.86	42.57
Cuba Cayman Islands	5	110790.50	95.90	25.05	32.59	16.73	1329.06	210.66	29.64
Cuyan Desaguadero	3	298283.30	1563.67	11.84	27.20	-2.53	265.88	42.98	7.74
Estero Real Tempisque	1	30035.90	303.12	25.46	33.29	18.74	1903.46	382.18	7.37
Florida Peninsula	1	156221.90	28.67	21.17	32.93	7.47	1307.71	191.27	51.95
Fluminense	4	11304.70	215.88	22.07	29.49	14.43	1282.51	191.19	40.01
Gila	1	159943.50	1179.89	16.21	35.04	-1.08	374.42	67.06	6.93
Grijalva Usumacinta	4	112525.90	471.12	24.22	33.10	15.14	1705.51	303.75	33.94
Guapore Itenez	1	354313.70	294.67	24.41	33.04	14.40	1321.92	223.60	15.46
Guianas	4	296984.10	147.43	26.06	32.56	21.09	2364.00	374.30	62.27
Guzman	5	132937.20	1588.24	15.37	33.62	-2.51	356.84	88.03	5.25
Iguassu	10	67537.70	795.45	17.26	27.50	6.27	1696.08	191.01	97.68
Isthmus Caribbean	1	10836.70	725.87	22.35	27.91	16.72	2998.48	405.96	100.69

Laguna dos Patos	5	181996.60	221.28	17.89	28.83	8.52	1419.89	140.46	94.40
Laurentian Great Lakes	1	763466.30	260.59	5.61	25.46	-14.3	848.34	95.16	43.39
Lerma Chapala	4	77443.80	2164.07	16.44	27.96	4.24	780.89	171.64	7.30
Llanos El Salado	2	67401.10	1871.58	17.90	30.45	4.52	427.02	78.14	7.58
Lower Mississippi	2	246389.70	72.17	17.35	32.99	0.65	1427.38	152.64	80.75
Lower Parana	27	643174.70	259.39	19.06	32.33	6.50	950.97	131.74	31.67
Lower Uruguay	7	270455.70	145.63	18.78	31.70	7.66	1394.18	147.80	87.40
Madeira Brazilian Shield	10	364797.80	187.19	25.20	33.23	16.91	2072.14	327.86	18.16
Magdalena Sinu	2	301018.70	1000.99	22.62	28.66	16.89	2035.57	289.74	52.95
Mamore Madre de Dios Piedmont	5	409221.50	249.86	25.41	32.87	16.81	2001.34	311.45	50.50
Mar Chiquita Salinas Grandes	4	567882.80	1317.57	14.84	28.92	0.44	432.73	75.43	7.55
Maracaibo	1	72114.90	681.06	24.16	30.11	17.65	1589.47	228.53	41.31
Mosquitia	1	123240.30	507.57	23.94	31.32	16.95	1900.81	287.06	41.00
North Andean Pacific Slopes Rio Atrato	2	261578.70	799.58	22.25	28.18	16.66	2535.88	352.90	101.17
Northeastern Caatinga & Coastal Drainages	6	286599.80	285.48	25.22	31.79	19.08	871.51	198.42	7.60
Northeastern Mata Atlantica	7	494883.20	483.68	22.49	29.89	14.56	1007.02	170.43	26.67
Orinoco Delta & Coastal Drainages	5	110486.90	105.14	26.10	31.64	20.70	1713.67	255.07	55.65
Orinoco Guiana Shield	3	301295.60	491.58	24.89	31.62	18.82	2660.82	422.49	57.94
Orinoco High Andes	1	56634.70	1864.32	17.37	23.40	11.35	2031.90	294.49	37.32
Orinoco Llanos	10	465952.50	123.49	27.17	34.56	21.42	2103.56	337.78	20.37
Orinoco Piedmont	3	73271.00	360.36	25.65	32.60	19.36	2234.27	326.37	27.71
Ouachita Highlands	1	115437.70	134.61	16.89	33.81	-0.93	1268.71	141.66	71.99
Papaloapan	1	56300.80	741.08	22.16	31.41	13.02	1759.88	354.96	29.52
Paraguay	10	546031.60	238.66	24.44	32.97	14.31	1393.20	216.83	30.05
Paraiba do Sul	4	64835.50	549.79	20.37	28.72	10.64	1395.75	249.53	23.43
Parnaiba	2	362472.00	329.62	25.99	34.05	18.27	1087.41	221.88	3.43
Puerto Rico Virgin Islands	1	9574.10	219.79	24.39	30.68	17.35	1761.89	228.28	64.57
Quintana Roo Motagua	4	96030.10	330.44	24.46	31.61	16.85	1667.92	277.22	41.73

Ribeira de Iguape	1	36616.70	523.14	19.53	27.97	10.05	1719.63	240.10	65.95
Rio Balsas	1	116232.00	1346.99	21.92	32.94	10.80	997.64	222.49	3.72
Rio Negro	7	496621.00	132.39	26.37	32.19	21.08	2857.97	361.19	143.63
Rio Santiago	4	93985.10	1692.23	18.47	30.47	5.44	779.43	202.25	4.01
S. Francisco	9	635926.90	630.72	23.35	31.55	13.99	957.10	197.43	3.95
Sabine Galveston	1	137327.90	103.09	18.80	34.36	2.10	1182.29	137.46	68.33
Sacramento San Joaquin	1	183455.80	803.02	12.70	31.33	-1.02	642.20	118.39	3.61
San Juan (Nicaragua/Costa Rica)	1	104659.50	245.80	24.87	31.25	18.95	2611.28	410.48	50.99
South America Caribbean Drainages Trinidad	1	133756.80	339.94	25.41	31.45	19.22	1024.73	177.70	16.03
South Andean Pacific Slopes	2	269554.10	1473.63	10.48	22.43	0.88	554.27	115.68	8.22
Southeastern Mata Atlantica	1	37710.50	407.03	18.62	27.66	9.67	1636.57	208.01	85.04
Teays Old Ohio	1	373583.90	332.66	10.96	29.21	-7.39	1096.94	117.07	66.68
Tocantins Araguaia	7	762552.80	370.68	25.53	33.42	17.00	1670.50	295.92	6.68
Tramandai Mampituba	1	8446.00	226.78	18.27	27.14	9.37	1494.59	157.98	97.23
Upper Parana	18	827360.90	613.72	21.68	29.85	11.38	1421.99	233.64	30.80
Upper Uruguay	3	78976.20	756.30	17.31	27.57	7.33	1792.02	185.78	119.93
Upper Usumacinta	2	65826.40	599.55	23.37	31.16	15.49	2184.96	358.72	51.32
US Southern Plains	1	417663.90	948.25	13.11	33.30	-7.07	578.54	92.10	16.19
Valdivian Lakes	1	59412.10	399.41	9.81	20.40	2.42	1909.61	296.53	61.98
West Florida Gulf	1	34434.00	76.40	18.61	32.64	2.89	1501.39	172.41	77.68
Yucatan	5	66922.80	37.01	25.80	34.50	16.58	1068.95	186.01	25.14

Table S3. Occurrence data on Neotropical diaptomids used for Parsimony Analysis of Endemicity. See table S4 for the names of each ecoregion.

Species name	0000000001111111111222222222233333333334444444444555555555566666666667777<br>1234567890123456789012345678901234567890123456789012345678901234567890123
Arctodiaptomus dampfi	0000000000000000000000000000000000000000000000000000000000000100000000000
Arctodiaptomus dorsalis	0000000000000000000000000000000000000000000000000101101111111111110000000
Arctodiaptomus dorsalis invasor	0000000000000000000000000000000000000000111111111100000000000000000000000
Argyrodiaptomus azevedoi	0000000000110001110000100000000000000000000000000000000000000000000000000
Argyrodiaptomus bergi	0000000000000000000000000000001100000010000000000000000000000000000000000
Argyrodiaptomus denticulatus	0000000000000000001000010000000111110000000000000000000000000000000000000
Argyrodiaptomus falcifer	0000000000000000000010010000000111000010000000000000000000000000000000000
Argyrodiaptomus furcatus	0000000000000000010000100101000100000000000000000000000000000000000000000
Argyrodiaptomus granulosus	0000000000000000000000000000000100000000000000000000000000000000000000000
Argyrodiaptomus macrochaetus	0000000000000000000000000000000100100000000000000000000000000000000000000
Argyrodiaptomus neglectus	0000000000000000100000000000000000000000000000000000000000000000000000000
Argyrodiaptomus nhumirim	0000000000000000000010000000000000000000000000000000000000000000000000000
Argyrodiaptomus paggii	0000000000001100000000000000000000000000000000000000000000000000000000000
Argyrodiaptomus robertsonae	0000000000100000000000000000000000000000000000000000000000000000000000000
Aglaodiaptomus clavipes	0000000000000000000000000000000000000000000000000000000000000000000000001
Aspinus acicularis	0000000000110000000000000000000000000000000000000000000000000000000000000
Austrinodiaptomus kleerekoperi	0000000000000000000000000000000000100000000000000000000000000000000000000
Calodiaptomus merrilae	0000000000101000001000000000000000000000000000000000000000000000000000000
Calodiaptomus perelegans	0000000000101000000000000000000000000000000000000000000000000000000000000
Colombodiaptomus brandorffi	0000000010000000000000000000000000000000000000000000000000000000000000000
Dactylodiaptomus pearsei	0000110010111000000000000000000000000000000000000000000000000000000000000
Dasydiaptomus coronatus	0000000010111100100000100001000100000000000000000000000000000000000000000
Diaptomus azureus	0000000000000000000000001100000000000000000000000000000000000000000000000
Diaptomus curvatus	0000000000000000000000000000000100000000000000000000000000000000000000000
Diaptomus fluminensis	0000000000000000000000000100000000000000000000000000000000000000000000000
Diaptomus frutosae	0000000000000000000000000000000100000000000000000000000000000000000000000
Diaptomus linus	0000000000100000000000000000000000000000000000000000000000000000000000000
Diaptomus negrensis	0000000001100000000000000000000000000000000000000000000000000000000000000
Diaptomus ohlei	0000000000100000000000000000000000000000000000000000000000000000000000000
Idiodiaptomus dussarti	0000000000000000000000010000000000000000000000000000000000000000000000000
Leptodiaptomus albuquerquensis	0000000000000000000000000000000000000000000000000000000000000000000000001
Idiodiaptomus gracilipes	0000000000000000000000110000000000000000000000000000000000000000000000000
Leptodiaptomus connexus	0000000000000000000000000000000000000000000000000000000000000000000000001
Leptodiaptomus cuauhtemoci	0000000000000000000000000000000000000000000000000000000100000000000000000
Leptodiaptomus dodsoni	0000000000000000000000000000000000000000000000000000000100100000000000000
Leptodiaptomus garciai	0000000000000000000000000000000000000000000000000000000010000000000000000
Leptodiaptomus novamexicanus	0000000000000000000000000000000000000000000000000000000000010000000000001
Leptodiaptomus patzcuarensis	0000000000000000000000000000000000000000000000000000000100000000000000000
Leptodiaptomus siciloides	0000000000000000000000000000000000000000000000000000001100000000000000001
Mastigodiaptomus albuquerquensis	0000000000000000000000000000000000000000000000000000001100010010000000000
Mastigodiaptomus asymmetricus	0000000000000000000000000000000000000000000000000000000000000010000000000
Mastigodiaptomus dorsalis	0000000000000000000000000000000000000000000000000000000000000010000000000
Mastigodiaptomus maya	0000000000000000000000000000000000000000000000000000000000010000000000000
Mastigodiaptomus montezumae	0000000000000000000000000000000000000000000000000000001100000000000000000
Mastigodiaptomus nesus	0000000000000000000000000000000000000000000000000000000000000110000000000
Mastigodiaptomus purpureus	0000000000000000000000000000000000000000000000000000000000000010000000000
Mastigodiaptomus reidae	0000000000000000000000000000000000000000000000000000000000010000000000000
Mastigodiaptomus suarezmoralesi	0000000000000000000000000000000000000000000000000000000000010000000000000
Mastigodiaptomus texensis	0000000000000000000000000000000000000000000000000000000000000110000000000
Notodiaptomus amazonicus	0000111000110001000000100001000100000000000000000000000000000000000000000
Notodiaptomus anisitsi	0000000000000000000010100001000101000000000000000000000000000000000000000
Notodiaptomus brandorffi	0000000000100001000000000000000000000000000000000000000000000000000000000
Notodiaptomus carteri	0000000000000000000000010000000100100000000000000000000000000000000000000
Notodiaptomus cearensis	0000000000000011110000101000000000000000000000000000000000000000000000000
Notodiaptomus conifer	0000000000000000000010110000000110010000000000000000000000000000000000000
Notodiaptomus coniferoides	1000000000111000001010110000000100000000000000000000000000000000000000000
Notodiaptomus dahli	0000000000010000000000000000000000000000000000000000000000000000000000000
Notodiaptomus deevoyrum	0001111001000000000010000000000100000000000000000000000000000000000000000
Notodiaptomus deitersi	0000000000000110001110110000000100000000000000000000000000000000000000000
Notodiaptomus dentatus	0000000000000000000000000000000100000000000000000000000000000000000000000
Notodiaptomus dilatatus	0000100000000000000000000000000000000000000000000000000000000000000000000
Notodiaptomus dubius	0000000000000000010000000000000000000000000000000000000000000000000000000
Notodiaptomus echinatus	0000100001000000000000000000000000000000000000000000000000000000000000000
Notodiaptomus gibber	0000000000000000000000000000100100000000000000000000000000000000000000000
Notodiaptomus henseni	0001110010110100000010100001000100000000000000000000000000000000000000000
Notodiaptomus iheringi	0000000000010001110000101001000000000000000000000000000000000000000000000
Notodiaptomus incompositus	0000000000000000000010000010111111100100000000000000000000000000000000000
Notodiaptomus inflatus	0000000010000000000000000000000000000000000000000000000000000000000000000
Notodiaptomus isabelae	0000000000000001100001000000000100000000000000000000000000000000000000000
Notodiaptomus jatobensis	0000000000100000100000100000000000000000000000000000000000000000000000000
Notodiaptomus kieferi	0000101000100000000000000000000000000000000000000000000000000000000000000

Notodiaptomus maracaibensis	110000000001010000000000000000000000000000000000000000000000000000000000
Notodiaptomus nordestinus	000000000000000100000000000000000000000000000000000000000000000000000000
Notodiaptomus paraensis	000000000010010000000000010000000000000000000000000000000000000000000000
Notodiaptomus pseudodubius	000000000100000000000000000000000000000000000000000000000000000000000000
Notodiaptomus santafesinus	000000000000000000001001000000010000000000000000000000000000000000000000
Notodiaptomus santaremensis	000000000011000000000000000000000000000000000000000000000000000000000000
Notodiaptomus similimus	000011000010000000000000000000000000000000000000000000000000000000000000
Notodiaptomus spiniger	000000000000000000000000000100110100000000000000000000000000000000000000
Notodiaptomus spinuliferus	000000000000010010001011000100010000000000000000000000000000000000000000
Notodiaptomus transitans	000000000000000000000011000000010100000000000000000000000000000000000000
Notodiaptomus venezolanus	010100000000000000000000000000000000000000000000000000000000000000000000
Odontodiaptomus michaelseni	000000000000000000000000000000010000000000000000000000000000000000000000
Odontodiaptomus paulistanus	000000000000000000000010101100000000000000000000000000000000000000000000
Odontodiaptomus thomseni	000000000000000000000000000000010100000000000000000000000000000000000000
Prionodiaptomus colombiensis	001010000000000000000000000000000000000000000000000000000101000001111110
Prionodiaptomus incarum	000000000000000000100000000000000000000000000000000000000000000000000000
Rhacodiaptomus besti	000000001010100000000000000000000000000000000000000000000000000000000000
Rhacodiaptomus calamensis	000000000010100000000000000000000000000000000000000000000000000000000000
Rhacodiaptomus calatus	000000001010100000000000000000000000000000000000000000000000000000000000
Rhacodiaptomus flexipes	000000000010000000000000000000000000000000000000000000000000000000000000
Rhacodiaptomus insolitus	000000000010100000000000000000000000000000000000000000000000000000000000
Rhacodiaptomus retroflexus	000000001010000000000000000000000000000000000000000000000000000000000000
Rhacodiaptomus ringueleti	000010000000000000000000000000000000000000000000000000000000000000000000
Scolodiaptomus corderoi	000000000000000011000010000000000000000000000000000000000000000000000000
Tumeodiaptomus diabolicus	000000000000000000000000000000000000110000000000000000000000000000000000
Tumeodiaptomus vivianae	000000000000000000000000000000000000010000000000000000000000000000000000
Hesperodiaptomus morelensis	000000000000000000000000000000000000000000000000000000100000000000000000

Table S4. Column names for the data matrix used for Parsimony Analysis of Endemicity indicated in Table X.

Column number	Ecoregion name
1	Magdalena Sinu
2	North Andean Pacific Slopes Rio Atrato
3	South American Caribbean Trinidad
4	Orinoco Piedmont
5	Orinoco Llanos
6	Orinoco Delta and Coastal Drainages
7	Orinoco Guiana Shield
8	Orinoco High Andes
9	Rio Negro
10	Guianas
11	Amazonas Lowlands
12	Amazonas Estuary and Coastal Drainages
13	Madeira Brazilian Shield
14	Tocantins Araguaia
15	Parnaiba
16	Northeastern Caatinga and Coastal Drainages
17	San Francisco
18	Northeastern Mata Atlantica
19	Mamore Madre de Dios Piedmont
20	Guapore Itenez
21	Paraguay
22	Amazonas High Andes
23	Upper Parana
24	Chaco
25	Paraiba do Sul
26	Fluminense
27	Ribeira do Iguape
28	Iguassu
29	Southeastern Mata Atlantica
30	Tramandai Mampituba
31	Upper Uruguay
32	Lower Parana
33	Mar Chiquita
34	Lower Uruguay
35	Laguna dos Patos
36	Cuyan Desaguadero
37	Valdivean Lakes
38	South Andean Pacific Slopes
39	Bonaerensean Drainages
40	Patagonia
41	Sacramento San Joaquin
42	Gila
43	US Southern Plains
44	Central Plains
45	Laurentian Great Lakes
46	Teays Old Ohio
47	Chesapeake Bay
48	Appalachian Piedmont
49	Ouachita Highlands
50	Lower Mississipi
51	Sabine Galveston
52	West Florida Gulf
53	Florida Peninsula
54	Llanos El Salado
55	Rio Santiago

56	Lerma Chapala
57	Rio Balsas
58	Papaloapan
59	Grijalva Usumacinta
60	Yucatán
61	Quintana Roo Motagua
62	Upper Usumacinta
63	Cuba Cayman Islands
64	Puerto Rico Virgin Islands
65	Estero Real Tempisque
66	Chiriqui
67	Chiapas
68	Mosquitia
69	San Juan Nicaragua
70	Istmus Caribean
71	Chagres
72	Maracaibo
73	Guzman Samalayuca
